# From plate to brain: role of mediators and moderators on the impact of diet on Alzheimer's disease

**DOI:** 10.3389/fnut.2026.1898935

**Published:** 2026-08-07

**Authors:** Hayley M. O'Neill, Kuldeep Kumar, Vatsana S. Rathore

**Affiliations:** 1 Faculty of Health Sciences and Medicine, Bond University, Gold Coast, QLD, Australia; 2 Centre for Data Analytics, Bond Business School, Bond University, Gold Coast, QLD, Australia

**Keywords:** brain health, cognitive health, dementia, dietary pattern, nutrition

## Abstract

Diet represents one of the most clinically accessible modifiable risk factors for Alzheimer's disease (AD), with potential to influence disease onset and progression through multiple biological pathways. Emerging evidence from observational studies suggests that adherence to specific dietary patterns, including the Mediterranean (MedDiet) and Mediterranean-DASH intervention for neurodegenerative delay (MIND) diets, is associated with reduced AD risk and slower cognitive decline. However, the mechanistic pathways underpinning these associations remain incompletely understood, and evidence from randomized controlled trials has been less consistent. A critical but frequently overlooked distinction lies between mediators, defined as the biological mechanisms linking diet to AD outcomes, and moderators, which are individual or contextual factors that influence the magnitude and direction of these associations. Integrating mediators and moderators within a unified analytical framework is increasingly recognized as essential for advancing precision nutrition approaches to AD prevention (with multiple recent publications explicitly calling for this approach and providing empirical examples of its implementation). This mini-review, developed as the conceptual foundation for a planned meta-analysis, synthesizes current evidence on key mediating pathways, including neuroinflammation, brain insulin resistance, oxidative stress, blood-brain barrier dysfunction, hyperhomocysteinaemia, and gut-brain axis dysregulation. We further examine major moderating factors such as apolipoprotein E (APOE) ε4 genotype, biological sex, age, disease stage, and socioeconomic context that may determine who benefits from dietary interventions and under what conditions.

## 1 Introduction

Alzheimer's disease (AD) and AD-related dementias are neurodegenerative disorders characterized by progressive decline in memory, cognitive function and the ability to perform daily activities.

Dementia is not a single disease but an umbrella term that encompasses a spectrum of brain disorders whose defining feature is progressive loss of cognitive function. This includes memory, reasoning, and the ability to manage everyday tasks as a result of neuronal damage and deterioration. While its prevalence increases with age, dementia can happen at any age. In 2021, ~ 50 million individuals over the age of 65 were living with dementia, a figure that has tripled from 1990 to 2021 and is further projected to triple by 2050 (1, 2). As the seventh leading cause of death globally, dementia is a major driver of disability and dependency in later life, with females consistently experiencing a disproportionately higher age-standardized burden in both disability-adjusted life years (DALYs) and mortality (1).

Of all the dementia subtypes, AD is the most common, responsible for roughly two-thirds of diagnoses in adults aged 65 and above. AD involves the aberrant processing and aggregation of amyloid-beta peptides into extracellular plaques. Combined with hyperphosphorylation of tau proteins into intraneuronal neurofibrillary tangles, these changes drive neuronal synaptic dysfunction and neuronal loss underlying clinical pathophysiology presentation of an insidious, progressive decline in cognition and behavioral function. Currently no disease-modifying cure exists.

AD has a strong genetic basis with up to 60%−80% of AD risk is attributable to genetic factors, with over 40 susceptibility loci identified to date; among these, variants in the APOE gene show the strongest association with disease risk (3). However, it is estimated that 40% of dementia could be prevented by 12 potentially modifiable risk factors, including education level, hearing loss, traumatic brain injury, alcohol consumption, hypertension, obesity, diabetes, smoking, depression, social isolation, physical inactivity, with risk associated with exposure increasing mainly either during midlife (4–24) or later life (>65) (25).

Prevention and early-risk reduction are emerging strategies to help alleviate symptom management. Diet is a modifiable lifestyle factor, and may play an important role in cognitive health through various pathways involved in vascular health and inflammation (26). In the last 10 years there has been a move toward whole dietary patterns (27), particularly high plant diets such as the Mediterranean diet (MedDiet) (28).

The MedDiet, traditionally followed by populations in the Mediterranean region (29). It is characterized by high composition of vegetables, legumes, fruits, whole grains, nuts, olive oil, and fish. It emphasizes low consumption of saturated fat, with moderate amounts of dairy products (primarily fermented sources such as cheese and yogurt), and limited intake of meat especially red and processed meat, as well as sweets and alcohol. This dietary pattern is one of the most extensively studied nutritional approaches and is widely recognized for its potential in the prevention and management of chronic health diseases, particularly in the primary and secondary prevention of CVD (30), all-cause mortality and emerging neurodegenerative conditions (28, 31). Whilst variations to MedDiet exist, the term reflects a variety of eating habits traditionally practiced by populations in countries bordering the Mediterranean Sea, with considerable variability by location (32). It provides abundant polyphenols, omega-3 fatty acids, and antioxidants to help reduce inflammation and improve vascular health. A recently meta-analysis involving 21 studies reports higher adherence to the MedDiet is association with an odds ratio of 0.89 (95% CI = 0.84–0.94) based on 65,955 participants and OR of 0.73 (95% CI = 0.62–0.85) in 38,292 participants with AD (26). Similarly, an alternative meta-analysis involving 23 studies showed the combined HR for cognitive impairment among those adhering to the MedDiet was 0.82 (95% CI 0.75–0.89); for dementia, the HR was 0.89 (95% CI 0.83–0.95); and for AD, the HR was 0.70 (95% CI 0.60–0.82), indicating substantial protective effects (33). In line with this, the WHO guidelines recommend a MedDiet to reduce the risk of cognitive decline or dementia, as it might help and does not harm (34).

The Dietary Approaches to Stop Hypertension (DASH) diet, originally developed to manage hypertension through an emphasis on fruits, vegetables, whole grains, and lean proteins alongside reduced saturated fat intake, has not demonstrated consistent cognitive benefits in prospective cohort studies. In the only relevant randomized trial, the DASH diet produced no independent improvements in memory, executive function, or global cognition, although benefits were observed when combined with aerobic exercise (28).

The MIND diet, which combines neuroprotective elements of the Mediterranean and DASH diets with a particular emphasis on leafy green vegetables, berries, and nuts, has shown promising associations with reduced risk of cognitive impairment in several observational cohort studies. However, a rigorous randomized controlled trial involving more than 600 older adults found no significant cognitive advantage of the MIND diet compared with a calorie-matched control diet after 3 years of follow-up (35).

Finally, dietary inflammatory potential, typically quantified using the Dietary Inflammatory Index, has demonstrated some of the most consistent epidemiological associations with dementia risk. All six identified observational studies reported that pro-inflammatory dietary patterns were associated with worse cognitive outcomes. Proteomic evidence further links inflammatory diet profiles to elevated AD biomarkers and neuroimaging markers of brain atrophy.

In contrast to these dietary patterns, Western diets consisting of higher levels of ultra-processed foods including refined carbohydrates, saturated and trans fats, and salt, are associated with elevated AD risk (36). These foods and dietary patterns appear to drive risk of insulin resistance, dyslipidemia, systemic inflammation, and cerebrovascular dysfunction. Moreover, increased meat consumption is associated with increased risk (37). More recently ketogenic diets and microbiome-targeted intervention have provided preliminary evidence as promising adjunct strategies. Additionally, meal timing interventions that induce metabolic switching, have also shown potential for promoting neuroplasticity and improving brain (38) and metabolic health (39).

Despite compelling evidence, the discipline consistently frames the diet-AD relationship as a direct association, neglecting the biological pathways through which diet exerts its effects (mediators) and the individual factors that shape those effects (moderators). Loughrey et al. (40) examined MedDiet adherence and cognitive health, and Ahmed, Zhang and Kumar (41) studied equilibrium dietary patterns between AD patients and healthy individuals, yet neither study formally modeled mediating or moderating variables. Addressing this gap is the central aim of the present review.

## 2 Conceptual framework: mediators and moderators

**Figure 1:**
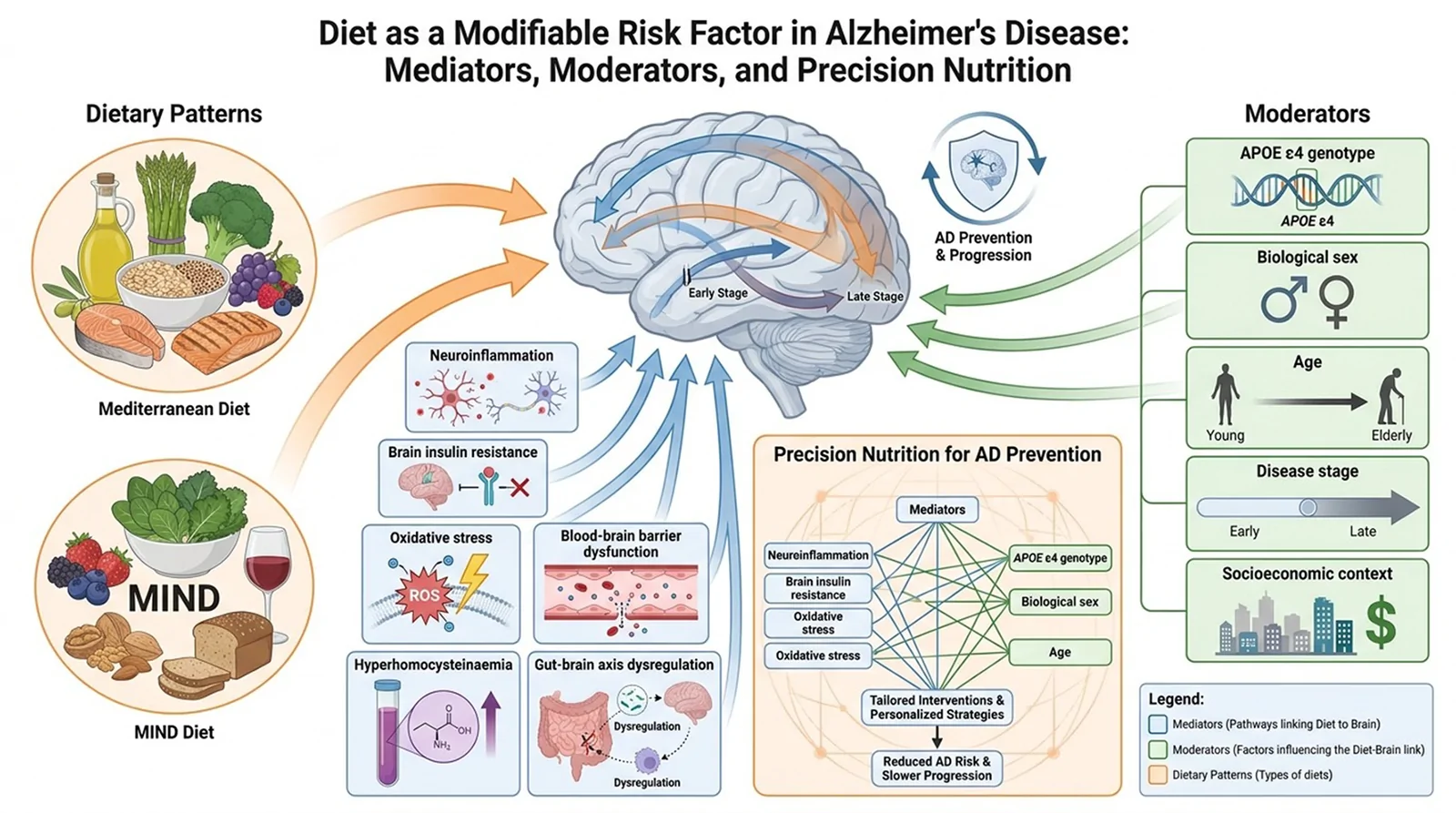
Diet as a modifiable risk factor for Alzheimer's disease. The effects of dietary patterns on AD risk and progression may operate through biological mediators, including neuroinflammation, insulin resistance, oxidative stress, blood-brain barrier dysfunction, hyperhomocysteinaemia, gut-brain axis dysregulation, and amyloid-β pathology, while being modified by factors such as APOE ε4 genotype, sex, age, disease stage, and socioeconomic context. Understanding these mediators and moderators may inform precision nutrition strategies for AD prevention and management.

This distinction between mediating mechanisms and moderating factors is more than semantic: a growing body of literature explicitly calls for their joint modeling as a foundation for prediction strategies for AD prevention (42–47). Mediators represent biological mechanisms along the causal pathway linking dietary exposures to AD outcomes, clarifying how and why diet influences neurological health. Through mediation analysis, the total effect of diet on AD can be partitioned into direct effects and indirect effects operating via specific biological processes, including neuroinflammation and inflammatory cytokines, insulin signaling, oxidative stress, gut microbiome-derived metabolites, and amyloid-β pathology (48). In contrast, moderators are factors that influence the magnitude or direction of the diet-AD association without being part of the causal pathway; key examples include APOE ε4 genotype, sex, age, and socioeconomic status (49). This distinction has important practical implications: identifying mediators elucidates underlying mechanisms and potential intervention targets, whereas identifying moderators informs the tailoring of dietary recommendations. A comprehensive approach that systematically incorporates both is critical for generating robust, individualized precision nutrition strategies that account for heterogeneity in both biological responses and disease risk pathways (Concept summarised in Figure 1).

## 3 Key mediating pathways

### 3.1 Neuroinflammation

Chronic neuroinflammation is a well-established contributor to AD pathogenesis (50, 51). The Western diet, typically rich in saturated fatty acids, activates toll-like receptor 4 (TLR4) via CD14/MD-2 co-receptor complexes, inducing NF-κB transcription and downstream expression of TNF-α, IL-1β, IL-6, and COX-2 (52). These inflammatory mediators cross or further compromise the blood-brain barrier (BBB), activating microglia and astrocytes in a feedforward cycle that accelerates Aβ aggregation (via BACE1 upregulation) and tau hyperphosphorylation through GSK-3β and CDK5 kinase activation (53). The NLRP3 inflammasome sits at the center of this neuroinflammatory phenotype and is nutritionally sensitive. For example, saturated fat and endotoxin prime NLRP3 activation, while olive oil-derived oleocanthal, omega-3 polyunsaturated fatty acids (PUFAs), and dietary polyphenols show inhibitory signatures in preclinical models (54). Mediterranean and Nordic dietary interventions reduce circulating CRP, IL-6, and TNF-α in human trials, providing mechanistic support for a neuroinflammatory mediation pathway (55). High-glycaemic diets further correlate with greater amyloid PET accumulation over time (56).

### 3.2 Insulin resistance and metabolic dysregulation

Insulin resistance (IR) and type 2 diabetes substantially accelerate Alzheimer's disease (AD), a relationship sufficiently robust to motivate the “type 3 diabetes” hypothesis. Insulin receptors are densely expressed in the hippocampus and prefrontal cortex, where insulin signaling supports amyloid-β (Aβ) clearance via insulin-degrading enzyme (IDE), promotes tau dephosphorylation, and maintains synaptic plasticity and neuronal survival (57). Under chronic hyperinsulinaemia, IDE is preferentially diverted toward circulating insulin, reducing its capacity to degrade Aβ (58). Concurrently, IR enhances GSK-3β and CDK5 activity, promoting tau hyperphosphorylation and neurofibrillary tangle formation, while activating NF-κB–mediated neuroinflammatory pathways.

Dietary patterns that promote peripheral insulin resistance, particularly high-glycaemic, ultra-processed Western diets rich in refined carbohydrates, saturated fats, and fructose—further impair brain insulin signaling through disruption of PI3K/Akt/mTOR pathways, thereby amplifying AD-related pathology. In contrast, low-glycaemic, fiber-rich Mediterranean-style diets attenuate these downstream metabolic and inflammatory pressures. Epidemiologically, the Rotterdam Study demonstrated that individuals with diabetes have nearly double the risk of developing AD (59), underscoring the clinical relevance of these mechanisms.

Therapeutically, metabolic interventions targeting impaired glucose utilization show emerging promise. Intranasal insulin has demonstrated modest improvements in verbal memory in early studies, while ketogenic diets provide ketone bodies (e.g., β-hydroxybutyrate) as an alternative cerebral fuel that bypasses impaired glucose metabolism. In addition to improving brain energetics, ketosis reduces insulin and IGF-1 signaling, suppresses mTOR, and activates AMPK. Preliminary trials in mild cognitive impairment (MCI) and early AD report improvements in cognitive outcomes and biomarker profiles, although these findings are derived from small, short-duration studies (60, 61). Collectively, insulin resistance represents a central mechanistic pathway linking diet to AD risk, particularly in populations with a high burden of metabolic syndrome.

### 3.3 Oxidative stress and advanced glycation end-products

Oxidative stress, characterized by an imbalance between reactive oxygen species (ROS) generation and antioxidant defense system, is a central feature of AD pathology and a mechanistically plausible dietary mediator (62). The brain is disproportionately vulnerable to oxidative damage given its high oxygen consumption, elevated polyunsaturated lipid content, and comparatively limited intrinsic antioxidant capacity. Western dietary patterns generate substantial oxidative load through reactive oxygen species (ROS) and advanced glycation end-products (AGEs). AGEs ligate RAGE receptors, activating NF-κB, impairing endothelium, and facilitating Aβ influx across the BBB. Elevated ROS promotes Aβ aggregation (via oxidative modification of amyloid precursor protein processing), tau oxidation and misfolding, mitochondrial dysfunction, and synaptic protein carbonylation (63).

Dietary polyphenols from berries, green leafy vegetables, extra-virgin olive oil, nuts, and dark chocolate are among the most potent inducers of endogenous antioxidant pathways, principally through Nrf2-ARE axis activation. Omega-3 PUFAs reduce lipid peroxidation by competing with arachidonic acid for cyclooxygenase substrates (64). Vitamin E and selenium from whole grains and nuts neutralize lipid peroxyl radicals. Epidemiological evidence consistently associates higher intakes of these antioxidant-rich foods, as highlighted in the MIND diet score, with lower blood and CSF markers of oxidative stress and with preserved cognitive function (65). Conversely, ultra-processed foods and refined carbohydrates promote glycation-mediated ROS and advanced glycation end -product (AGE) accumulation, both documented contributors to AD-related neurodegeneration (4, 5).

### 3.4 Blood-brain barrier integrity and cerebrovascular function

The blood-brain barrier (BBB) governs the entry of nutrients, metabolites, and potentially harmful molecules into the brain parenchyma. BBB dysfunction is now recognized as an early event in AD pathogenesis, preceding Aβ plaque deposition in some imaging studies, and is mechanistically linked to pericyte loss, tight junction protein degradation, and neuroinflammation-driven endothelial activation. Diet influences BBB integrity through multiple mediating pathways: inflammatory cytokines induced by Western dietary patterns disrupt tight junction proteins (ZO-1, occludin, claudin-5); LPS derived from gut dysbiosis further compromises endothelial barrier function; oxidative stress promotes lipid peroxidation of membrane phospholipids in brain endothelial cells.

Conversely, omega-3 PUFAs, polyphenols, and plant-derived flavonoids support endothelial function through eNOS activation, nitric oxide bioavailability, and anti-inflammatory signaling. Adherence to the MedDiet and MIND diets has been associated with improved cerebrovascular integrity on neuroimaging and with reduced white matter hypervolume, a surrogate for vascular contributions to cognitive impairment (6). This vascular-BBB pathway may be especially relevant as a mediator in populations with comorbid hypertension, metabolic syndrome, or cardiovascular disease.

Cerebrovascular dysfunction represents a critical pathway in which dietary patterns may influence AD pathogenesis and cognitive outcomes. Neurovascular dysfunction occurs early in AD pathological process and may serve as both an initiator and accelerator of AD pathology (7). Reduced cerebral perfusion is among the earliest detectable alterations in individuals with AD, and impairs amyloid clearance, contributing to pathological accumulation. Dietary patterns exert protective effects through multiple vascular mechanisms: the DASH and Mediterranean diets reduce hypertension and hyperglycaemia through lower sodium and higher potassium, fiber, and unsaturated fat intake, thereby improving endothelial function and glycaemic control. Targeting these modifiable cardiovascular risk factors through dietary patterns such as the Mediterranean and DASH diets may confer neuroprotective benefits by improving vascular and metabolic function, thereby preserving cerebrovascular integrity and aligning dementia prevention strategies with established cardiovascular disease prevention frameworks.

Elevated homocysteine is among the most actionable dietary mediators: it promotes amyloidogenic APP processing, tau phosphorylation, endothelial injury, and oxidative stress, and is directly modifiable through dietary B-vitamin intake. The VITACOG trial demonstrated that B-vitamin supplementation slows brain atrophy in MCI, with cognitive benefit most evident when omega-3 status is simultaneously adequate, illustrating a mediator-mediator interaction requiring explicit modeling (8).

### 3.5 Gut-brain axis

The gut microbiome serves as a critical interface between dietary patterns and brain health. Plant-based, high-fiber diets selectively enrich short-chain fatty acid (SCFA)-producing microbial taxa; butyrate, a principal SCFA, confers neuroprotection through histone deacetylase (HDAC) inhibition. In contrast, gut dysbiosis facilitates lipopolysaccharide (LPS) translocation into systemic circulation, activating TLR4 signaling on brain endothelial cells and microglia. Additionally, the gut-derived metabolite trimethylamine N-oxide (TMAO), which is biosynthesised from dietary choline and carnitine by colonic bacteria, can traverse the blood-brain barrier (BBB) and induces astrocytic neuroinflammation (9, 12).

Compositional and diversity-level alterations in the gut microbiome have been directly implicated in AD pathology. Fecal analyses of individuals with AD reveal reduced microbial diversity alongside a compositionally distinct microbiota relative to cognitively healthy controls (11). Critically, these microbiome differences correlate with cerebrospinal fluid (CSF) tau levels and plasma amyloid-β (Aβ) biomarker concentrations at early disease stages, suggesting that gut dysbiosis may reflect or reinforce upstream neuropathological processes (11). Specifically, AD is characterized by gut microbiota dysbiosis with depletion of beneficial SCFA-producing bacteria (*Faecalibacterium prausnitzii, Roseburia, Lachnospiraceae, Eubacterium*) and enrichment of pro-inflammatory Proteobacteria (*Escherichia/Shigella, Enterobacteriaceae*), a pattern that Mediterranean and ketogenic dietary interventions can favorably reverse to improve microbiome composition (10, 12).

Intestinal barrier disruption appears to be a key mechanistic link between gut dysbiosis and cognitive decline. Individuals with cognitive impairment exhibit elevated circulating inflammatory markers including IL-6, IL-1β, LPS-binding protein (LBP), and TLR4, alongside reduced plasma zonula occludens-1 (ZO-1), a marker of tight junction integrity. Elevated fecal calprotectin further corroborates heightened intestinal inflammation in this population (13). Collectively, these findings indicate that compromised gut barrier function and systemic inflammatory signaling may substantially contribute to the trajectory of cognitive decline. Despite this and the considerable attention to Mediterranean, DASH and MIND diets for AD, the role of gut microbiota as a mediating pathway remains poorly understood (48). The neuroprotective effects of diet are largely attributed to anti-inflammatory mechanisms mediated by microbial metabolites of dietary fiber and polyphenols (14). In prospective cohort data, greater adherence to the MedDiet was associated with a more favorable gut microbiota profile (ie. enrichment of SCFA-producing bacteria (particularly *Barnesiella* and *Butyricicoccus*) and depletion of pro-inflammatory *Eggerthella*, forming a 20-taxa MedDiet gut microbial signature) and attenuated cognitive decline over 6 years of follow-up (15). Conversely, Western dietary patterns, characterized by high sugar and saturated fat content, disrupt microbial homeostasis, increase intestinal permeability, and promote systemic inflammation, oxidative stress, and lipid metabolic dysregulation, collectively accelerating neurodegeneration (16). Beyond diet, emerging evidence suggests that sleep and physical activity also modulate the gut microbiome–AD relationship and warrant consideration as integrated lifestyle determinants of disease risk (10).

### 3.6 Methodological priorities for mediation analysis

Robust mediation analysis in nutrition-AD research requires: (i) temporal ordering where diet precedes mediator, mediator precedes outcome, ideally with repeated measures; (ii) counterfactual causal mediation or SEM frameworks to estimate the indirect effect (a × b) with bootstrapped confidence intervals; (iii) pre-specification of confounders (age, sex, education, vascular risks, APOE genotype) and moderators; (iv) validated mediator assays (CSF sPDGFRβ or (dynamic contrast enhanced MRI (DCE-MRI) for BBB; longitudinal PET SUVR for amyloid; enzymatic/LC-MS for homocysteine); and (v) reporting of the proportion of total effect mediated by each pathway to enable cross-study synthesis.

## 4 Key moderating pathways

### 4.1 APOE ε4 genotype

Apolipoprotein E (ApoE) is known as a key regulator of lipid transport and cholesterol homeostasis in the brain. It is primarily produced by astrocytes and microglia, with additional contributions from neurons. Of its three isoforms (ApoE2, ApoE3 and ApoE4), ApoE4 The APOE ε4 allele is the strongest genetic risk factor for late-onset AD, increasing lifetime risk up to 15-fold compared to ε3 homozygotes(17). Structurally less stable, ApoE4 exhibits impaired lipid binding and reduced amyloid-β clearance, while also promoting tau hyperphosphorylation, neuroinflammation, and oxidative stress. Beyond classical pathology, ApoE4 disrupts mitochondrial function exacerbates lipid oxidation, and impairs insulin signaling, contributing to early deficits in brain glucose metabolism, an emerging metabolic pathway linked to accelerated cognitive decline (18).

Some cohort analyses including Memory and Aging Project (MAP) and Rush Memory and Aging Project (581, 73% women), demonstrate that the protective effect of Mediterranean-style patterns is stronger in ε3/ε3 non-carriers than ε4 carriers (19), while preclinical data (including APOE4 knock-in, APP/PS1/APOE-ε4, and Female ApoE4 knock-in mice) showing that Western diet-induced neuroinflammation is substantially amplified by the APOE4 allele (19–21, 30). Specifically, in exploratory analyses from these human cohorts, only APOE-ε4 non-carriers showed significant benefit with higher green leafy vegetable and beans intake and reduced intake of fried/fast foods and pastries/sweets (22). Conversely, ε4 carriers may be more metabolically responsive to dietary fat reduction, potentially identifying them as a priority subgroup. However, more recent data presents a complex and evolving picture. The Nurses' Health Study (4,215 women) and Health Professionals Follow-up Study (1,490 men) found that adherence to the MedDiet more effectively modulated dementia-related metabolites in APOE4 homozygotes, suggesting targeted prevention strategies may actually be more beneficial in this high-risk genetic group (43). As such, APOE genotype must be treated as a primary moderator, not merely a covariate, in future stratified analyses and clinical trials powered to detect genotype-by-diet interaction effects (18). In addition to APOE gene, a number of other genetic variants have been associated with AD (3, 17). The concept of APOE-dependent metabolic alterations is further supported by Arnold et al. (2020), which found substantial sex and APOE ε4 genotype differences in metabolic effects, with several group-specific alterations not observed in unstratified analyses (23).

### 4.2 Biological sex

Approximately two-thirds of AD cases occur in females, yet the interaction between sex and dietary exposures remains poorly characterized. The female reproductive hormone estrogen is known to activate neuroprotective signaling cascades including upregulation of brain-derived neurotrophic factor (BDNF), PI3K/Akt and MAPK/ERK pathways, and promotes β-amyloid clearance through multiple mechanisms. Estrogen-mediated effects also inhibit glycogen synthase kinase 3-beta, thereby reducing tau hyperphosphorylation, and upregulating anti-inflammatory signaling in astrocytes and microglia. These neuroprotective effects operate through both genomic (via estrogen receptors ER α and Erβ) and non-genomic mechanisms, with cross-talk between estrogen and BDNF signaling creating synergistic protective effects. Women demonstrate better brain resilience to tau pathology but paradoxically have higher tau loads despite similar amyloid burden (24, 66).

In preclinical models, female APOE4 mice show the most severe metabolic and neuroinflammatory phenotypes in response to Western diet, with compounding effects of genotype and sex on AD pathology (67, 68). Evidence from MIND diet cohorts suggests differential associations with cognitive decline by sex and race (Agarwal et al.,). Most existing trials are underpowered to detect sex-by-diet interactions; pre-registering sex as a primary moderator is essential in future study designs.

Emerging evidence demonstrates that dietary interventions exert sex-specific effects on AD pathology and cognitive outcomes, suggesting variable metabolic responses to different foods between women and men (22). For example, in the REGARDS cohort involving ~17,700 participants, greater MIND diet adherence was associated with reduced risk of incident cognitive impairment in women but not in men (sex interaction *p* < 0.05) (69). Similarly, although greater MIND diet adherence was associated with slower cognitive decline in both sexes, the association was significantly stronger in women (69). These findings may reflect differential effects of diet on cognitive reserve in women and men, though most previous studies have not been powered to detect sex-by-diet interactions.

Food-specific sex differences have also been identified in autopsy studies examining diet-AD pathology relationships. Women with higher green leafy vegetable intake, lower red and processed meat consumption, and recommended wine intake (1 glass per week) exhibited less AD pathology, including reduced amyloid plaque burden (70). By contrast, men with higher fish intake and moderate poultry consumption had less AD pathology. Unexpectedly, men with recommended wine intake and higher nut consumption showed greater phosphorylated tau (70) burden (70); however, these exploratory findings did not survive correction for multiple comparisons. Collectively, these observations suggest sex-specific metabolic or neurobiological responses to dietary exposures that warrant further investigation in precision nutrition frameworks.

The menopausal transition represents a critical window during which declining estrogen levels precipitate metabolic and neurobiological changes relevant to AD risk. Neuroimaging studies demonstrate that perimenopausal and postmenopausal women exhibit increased brain hypometabolism, greater amyloid-β deposition, elevated tau burden (71), and structural brain loss compared with premenopausal women and age-matched men (72). Loss of estrogenic regulation of cerebral bioenergetics induces a hypometabolic state (73), potentially triggering compensatory reliance on ketone metabolism and white matter catabolism, thereby accelerating neurodegenerative processes.

Collectively, these data support a model in which sex modifies diet-AD associations through intersecting pathways involving hormonal regulation, brain metabolism, immune function, and genetic susceptibility. Future studies should pre-register sex as a primary moderator, incorporate hormonal and reproductive staging, and examine sex-specific dietary responses across the lifespan. Such approaches are essential to advance precision nutrition strategies that account for differential vulnerability to obesogenic diets and optimize AD prevention in both women and men.

### 4.3 Age, disease stage, and comorbidities

Age moderates the diet-AD relationship because biological aging alters the efficiency of mediating pathways and because AD pathology evolves decades before clinical symptom onset. Age is the most important risk factor for both AD and dementia (17). Younger individuals show better brain resilience to tau pathology, maintaining structural integrity despite similar tau burden (24).

Age, particularly at the time of dietary assessment, represents a key moderating for consideration. In a recent systematic review involving 64 studies of 141 dietary patterns, Yusufov et al. noted that 81% of studies included participants with mean baseline ages exceeding 65 years, placing them within the established at-risk window for AD. This limits the ability to determine whether dietary patterns exert their greatest influence earlier, during the long preclinical phase of disease development (74).

Drawing on data from the Washington Heights–Inwood Columbia Aging Project (WHICAP), 2,258 community-dwelling individuals without dementia were followed prospectively, with assessments conducted every 1.5 years over a mean follow-up of 4 years (range 0.2–13.9 years) (75). During this period, 262 participants developed incident AD (75). Scarmeas et al. were among the first to demonstrate a protective association between adherence to the MedDiet and the risk of developing AD (75). However, this cohort similarly enrolled adults >65 years of age leaving the question of benefits of earlier dietary windows of interventions unaddressed.

At the intervention level, the FINGER (Finnish Geriatric Intervention Study to Prevent Cognitive Impairment and Disability) trial was the first large scale RCT to demonstrate a significant effect of multidomain lifestyle intervention on cognitive decline, enrolling 1,260 older adults (ages 60–77 years of age) with elevated dementia risk (76). In this study, diet comprised one of the four intervention components. After 2 years, global cognitive function was ~25% higher in the intervention group, suggesting that dietary change alongside other lifestyle modification retained meaningful impact even in later life (76). Nevertheless, whether earlier intervention confers greater benefit remained unresolved.

Glans et al. directly addressed the age-at-measurement limitation by prospectively assessing midlife dietary habits over a 20-year follow-up, yet found no significant associations between adherence to either conventional dietary recommendations or a modified MedDiet and subsequent risk of all-cause dementia, AD, or vascular dementia (77). Interpreted alongside the methodological concerns raised by Yusufov et al., including the risk of reverse causality when participants older than 70 are recruited, given that undetected cognitive impairment may itself alter dietary behavior, these findings collectively underscore that age as a moderator of the diet–AD relationship remains an open empirical question, and that the field requires studies with broader age ranges and longer follow-up periods to determine whether a critical dietary intervention window exists within the preclinical phase of AD.

Midlife has emerged as a critical period for nutritional assessment and intervention to prevent cognitive decline. Findings from UK Biobank and CARDIA studies demonstrate with the strongest evidence showing that dietary patterns followed during ages 45–54 years have the most pronounced associations with later cognitive outcomes (78, 79). Moreover, obesity and systemic inflammation are linked to cognitive function in midlife adults (2).

### 4.4 Physical activity

Physical activity is a lifestyle moderator that can amplify dietary effects on cognitive health (80). Exercise independently activates BDNF-mediated neuroplasticity through multiple pathways, including muscle-derived myokines (cathepsin B, irisin/FNDC5) that cross the BBB and upregulate BDNF expression in the hippocampus (81). Physical activity also reduces neuroinflammation via microglial modulation and enhances insulin sensitivity in both peripheral and central nervous systems, overlapping substantially with dietary mediating pathways (81). Meta-analyses of prospective cohort studies consistently show that physical activity is associated with reduced risk of cognitive decline. The most comprehensive meta-analysis (*n* = 257,983 participants from 58 studies) found that physical activity was associated with a 20% reduced risk of all-cause dementia (pooled RR 0.80, 95% CI 0.77–0.84) and 14% reduced risk of AD specifically (RR 0.86, 95% CI 0.80–0.93). A recent dose-response meta-analysis (*n* = 1,453,561 participants, 68,497 AD cases) demonstrated that high-intensity physical activity reduced AD risk by 26% (HR 0.74, 95% CI 0.67–0.83), with linear dose-response analyses showing a 15% reduction in AD risk for every 10 MET-hours/week increase in physical activity (82). The protective effect was more pronounced in non-obese individuals (BMI < 25; HR 0.65, 95% CI 0.52–0.82), those aged ≥75 years (HR 0.57, 95% CI 0.48–0.67), and non-APOE ε4 carriers (HR 0.72, 95% CI 0.55–0.93) (82).

Whether combining exercise with dietary modifications further improves outcomes has also been explored. Recent meta-analyses examining combined diet and exercise interventions demonstrate modest benefits on global cognition (SMD 0.15–0.32), with the strongest effects observed in individuals at risk for cognitive decline rather than cognitively healthy populations, and combined interventions may be superior to single-domain approaches in some trials (83, 84).

Multi-domain interventions including (US POINTER, FINGER, MAPT, MIND-ADmini) all embed dietary modifications within exercise and cognitive training frameworks. The US POINTER trial (involving over 1,000 participants in each group, 60–79 years, 2-year duration intervention) demonstrated that a structured multidomain intervention including diet, exercise, cognitive training, and vascular risk management produced statistically significant but modest cognitive benefits (difference of 0.029 SD per year) compared to self-guided intervention (85). The FINGER trial showed benefits on executive function and processing speed in at-risk older adults (76). The MAPT trial did not show significant effects on the primary cognitive outcome overall, though exploratory subgroup analyses suggested benefits in individuals with positive amyloid status or higher dementia risk scores (86, 87). The MIND-ADmini pilot trial demonstrated feasibility and improved dietary quality in individuals with prodromal Alzheimer's disease, though cognitive outcomes from this 6-month pilot remain to be fully reported (46, 47).

### 4.5 Socioeconomic context

Socioeconomic status (SES), food security, neighborhood food environment, and cultural dietary norms all moderate whether individuals can adopt and sustain protective dietary patterns like the MedDiet (88–90). Lower household income is consistently associated with lower adherence to Mediterranean and MIND diets, and neighborhood socioeconomic characteristics shape dietary adherence independent of individual-level factors (89–91). Importantly, SES modifies the diet-cognition relationship itself: higher adherence to the MedDiet diet was associated with reduced cognitive decline only among high-SES individuals in Italian cohorts, while no association was observed in low-SES groups (90, 92). This suggests that equal dietary adherence may confer differential cognitive benefits depending on socioeconomic context.

### 4.6 Other moderating factors

Race and ethnicity moderate both dietary exposure and the diet-cognition relationship. In the Multiethnic Cohort Study, protective associations of Mediterranean, DASH, and MIND diets with dementia risk were stronger in African American, Latino, and White participants than in Japanese American and Native Hawaiian participants (93). The MIND diet showed protective associations in both Black and White older adults in the Chicago Health and Aging Project, though the association in Black participants was attenuated by vascular and lifestyle factors (93). In the REGARDS (REasons for Geographic and Racial Differences in Stroke, dietary data from 14,145 participants, ~64 ± 9 years years) cohort study, demonstrated that MIND diet adherence was a stronger predictor of cognitive decline in Black participants than in White participants (93). These findings indicate that dietary indices developed in predominantly European populations may inadequately capture protective patterns in other cultural contexts (39, 93). Meta-analyses must pool across diverse cohorts with adequate socioeconomic characterization and report ethnicity-stratified estimates to generate globally applicable dietary guidance.

## 5 Toward an integrated mediator-moderator framework

The evidence presented in this review calls for an integrative conceptual model in which dietary patterns operate through multiple, partially overlapping biological pathways (ie. inflammation, oxidative stress, etc) with the magnitudes and clinical relevance of these effects shaped by APOE genotype, biological sex, age, physical activity levels, presence of comorbidities, and socioeconomic status among others modifiable risk factors previously identified.

The novelty in the moderator-mediator framework is in the degree to which the empirical literature can provide mechanistic content for each component of the framework. Genetic factors, particularly APOE genotype, moderate dietary effects on cognitive outcomes, with evidence suggesting that APOE ε4 carriers and non-carriers may respond differently to dietary interventions (42, 43, 94). Recently, this same Nurses' Health Study/Health Professionals Follow-up Study cohort provided further mechanistic detail: cholesteryl esters and sphingomyelins were identified as the metabolites most strongly associated with increased dementia risk among APOE ε4 homozygotes (43).

Recently, integration of genetic, plasma metabolomic and dietary data from 4,215 women and 1,490 men from prospective cohorts highlighted that adherence to the MedDiet was more effectively modulated dementia-related metabolites in APOE ε4 homozygotes (43). Specifically, cholesteryl esters and sphingomyelins were most strongly associated with increased dementia risk in this genetic subgroup (43). Similarly, APOE genotype modulates the estimated effects of dietary macronutrients on cognitive performance, with higher protein and lower carbohydrate/fat ratios showing relatively more favorable associations with cognition at increasing levels of APOE ε4 risk (42). However, the overall evidence remains mixed with some studies show greater dietary benefits in APOE ε4 non-carriers, while others suggest Western dietary patterns increase dementia risk specifically among ε4 carriers (22, 42).

Moreover, such a framework also has direct implications for meta-analytic methodology. Pooling crude diet-AD associations across studies that differ in participant genotypes, ages, sexes, race, chronic disease status and socioeconomic compositions could systematically affect heterogeneity and may underestimate effect sizes in subgroups where effects are large while overestimating them in others (39, 69, 93, 95). Current meta-analyses face substantial methodological challenges, including high risk of bias in original studies, inappropriate synthesis methods, inadequate assessment of primary study quality, and heterogeneity in dietary assessment and adherence tools and dementia diagnostic criteria (77, 95–97). An umbrella review of 20 meta-analyses of prospective studies investigating dietary factors and neurodegenerative disorders found that all included meta-analyses were rated as being at high risk of bias, with methodological concerns relating mainly to inappropriate synthesis, assessment, and discussion of the risk of bias of primary studies (95).

Whilst existing meta-analyses have established that protective dietary patterns like the MedDiet, MIND and DASH diets reduce dementia risk; however, the next generation must quantify for whom and through what pathway those effects are largest. This requires individual participant data (IPD) meta-analyses with pre-specified moderator analyses stratified by genetic risk, age, sex, and socioeconomic factors, as well as biomarker-enriched sub-studies that allow estimation of mediated effects using causal mediation analysis frameworks (43). Recent evidence demonstrates the feasibility of this approach: ethnicity-stratified analyses in the Multiethnic Cohort Study revealed that protective associations of Mediterranean, DASH, and MIND diets with dementia risk varied substantially across racial and ethnic groups (93), while socioeconomic stratification showed that higher MedDiet adherence reduced cognitive decline only among high-SES individuals in Italian cohorts (90).

Two-sample Mendelian randomization (MR), which uses genetic instruments for dietary intake components to estimate causal effects on outcomes, represents a powerful tool for establishing whether observed associations are causal rather than confounded (98–102). However, current MR evidence yields mixed results. Some studies support causal protective effects of higher relative protein and fat intake on AD risk, while others find causal relationships between processed meat, poultry, and beef consumption and increased AD risk (99, 100). Notably, a recent study using UK Biobank GWAS data from 361,194 participants found no statistically significant causal associations between 20 dietary habits and AD risk, highlighting the challenges of using genetic instruments for complex dietary behaviors (98). Two-step MR with mediation analysis, which uses genetic instruments for dietary components to estimate causal effects on mediating biomarkers, then on cognitive outcomes, has been applied to gut microbiota and metabolites, identifying specific mediation pathways (e.g., homostachydrine mediating 39% of the effect of genus Turicibacter on emotion recognition) (9, 103). This approach appears to be promising for dietary research but requires validation.

Integration of multi-omic data (metabolomics, microbiomics, proteomics) across dietary intervention studies is enabling identification of nutriome-cognitive outcome relationships with extraordinary mechanistic resolution (9, 104). Recently, prospective study involving 746 participants ~65 years of age demonstrated that a gut microbial signature of MedDiet adherence (comprising 20 taxa including short-chain fatty acid producers) was independently associated with slower global cognitive decline and preserved executive function over 6 years (15).

Metabolomic profiling identified 19 putative causal relationships between metabolites and cognitive outcomes, including protective effects of 4-guanidinobutanoate, carotenoids, and specific nucleosides (43). Importantly, the associations of 57 metabolites with dementia risk varied by APOE4 genotype or other AD genetic risk variants, demonstrating genotype-dependent metabolic profiles of cognitive health (43). This prospective study integrated genetic, plasma metabolomic, and dietary data from 4,215 women and 1,490 men in prospective cohorts, making it one of the most comprehensive investigations of genotype-dependent metabolic profiles in cognitive health to date.

These multi-omic approaches are starting to reveal that diet influences cognition through multiple pathways including microbial metabolites (short-chain fatty acids, tryptophan metabolites, bile acids), inflammatory markers, and lipid metabolism, providing mechanistic support for precision nutrition strategies tailored to individual genetic and metabolic profiles (15, 105).

### 5.1 Limitations and future direction

Several methodological limitations constrain current inferences. Dietary assessment relies predominantly on self-reported food frequency questionnaires subject to recall bias; objective dietary biomarkers (plasma carotenoids, omega-3 index, urinary flavonoid metabolites) should be incorporated as validity anchors. Outcome heterogeneity across studies including neuropsychological scores, clinical diagnosis, and PET/CSF biomarker staging can complicate pooling; harmonized outcomes aligned with the revised Alzheimer's Association ATNIVS biological staging framework are needed. Most dietary intervention RCTs (12–24 months) are fundamentally mismatched to the multi-decade timescale of AD pathological evolution, limiting causal inference. Evidence from low- and middle-income country settings, where the majority of projected AD burden growth will occur, remains critically sparse, and culturally adapted dietary indices for AD prevention represent a priority research area. For the planned meta-analysis, individual participant data (IPD) pooling with pre-specified moderator stratification, and two-step Mendelian randomization to establish causal directionality of mediating pathways, represent the highest methodological priorities.

## 6 Conclusions

Diet-AD relationships are driven by interacting pathways, including neuroinflammation, insulin resistance, oxidative stress, BBB dysfunction, homocysteine, and the gut-brain axis, modulated by genotype, sex, age, lifestyle, and context. Ignoring this complexity limits actionable guidance. Current evidence supports Mediterranean, MIND, and DASH diets for neuroprotection, while Western patterns increase risk; ketogenic, and microbiome-based approaches remain promising but undertested. Progress will require precision nutrition frameworks integrating causal mediation, biomarkers, and stratified analyses to deliver mechanistically grounded, individualized recommendations.
